# Effect of elective cesarean section on the risk of mother-to-child transmission of hepatitis B virus

**DOI:** 10.1186/1471-2393-13-119

**Published:** 2013-05-24

**Authors:** Yali Hu, Jie Chen, Jian Wen, Chenyu Xu, Shu Zhang, Biyun Xu, Yi-Hua Zhou

**Affiliations:** 1 Department of Obstetrics and Gynecology, Nanjing Drum Tower Hospital, Nanjing Medical University, Nanjing, China; 2 Department of Obstetrics and Gynecology, Zhenjiang Fourth People's Hospital, Zhenjiang, China; 3 Department of Biostatistics, Nanjing Drum Tower Hospital, Nanjing Medical University, Nanjing, China; 4 Departments of Laboratory Medicine and Infectious Diseases, Nanjing Drum Tower Hospital, Nanjing Medical University, Nanjing, China; 5 Jiangsu Key Laboratory for Molecular Medicine, Nanjing University Medical School, Nanjing, China

**Keywords:** Hepatitis B virus, Mother-to-child transmission, Vaginal delivery, Caesarean section

## Abstract

**Background:**

Many clinicians and hepatitis B virus (HBV)-infected pregnant women prefer elective caesarean section (ECS) to prevent mother-to-child transmission of HBV, since some studies found higher transmission of HBV in infants born by vaginal delivery (VD) than by cesarean section. However, other studies showed that ECS does not reduce the risk of being infected with HBV in infants. In this study, we aimed to clarify whether ECS may reduce the risk of mother-to-child transmission of HBV.

**Methods:**

Totally 546 children (1–7-year-old) born to 544 HBsAg-positive mothers from 15 cities and rural areas across Jiangsu Province, China, were enrolled. Of these children, 137 (2 pairs of twins) were born to HBeAg-positive mothers; 285 were delivered by ECS and 261 others by VD (one pair of twin in each group). HBV serologic markers were tested by enzyme or microparticle immunoassay.

**Results:**

The maternal and gestational ages, maternal HBeAg-positive rates, and children’s ages, gender ratios, hepatitis B vaccine coverage and administrations of HBIG were comparable between ECS and VD groups (all p >0.05). The overall prevalence of HBsAg in the 546 children was 2.4%, with 2.5% (7/285) and 2.3% (6/261) in those born by ECS and VD respectively (p = 0.904). Further comparison of chronic HBV infection in the 137 children of HBeAg-positive mothers showed that the HBsAg-positive rates in ECS and VD groups were 10.3% (7/68) and 8.7% (6/69) respectively (p = 0.750), while the mothers had similar HBV DNA levels (2.38 × 10^6^ vs. 2.35 × 10^6^ IU/ml, p = 0.586). Additionally, the overall rate of anti-HBs ≥10 mIU/ml in the children was 71.6%, with 72.3% and 70.9% in those born by ECS and VD respectively (p = 0.717).

**Conclusions:**

With the recommended immunoprophylaxis against hepatitis B, ECS does not reduce the risk of mother-to-child transmission of HBV. Therefore, ECS should not be used in HBsAg-positive pregnant women to prevent mother-to-child transmission of HBV.

## Background

Hepatitis B virus (HBV) infection remains a major health threat worldwide; each year over one million individuals die from HBV-related diseases, including cirrhosis and hepatocellular carcinoma. Mother-to-child transmission of HBV has been recognized as the major cause of chronic HBV infection, particularly in highly endemic areas such as Southeast Asia and Africa [1]. Therefore, prevention of mother-to-child transmission of HBV is critical to control the infection. The recommended immunoprophylaxis against hepatitis B, including use of hepatitis B vaccine and hepatitis B immune globulin (HBIG) in infants, has been proven to be the most effective way to reduce perinatal HBV transmission.

Mother-to-child transmission of HBV occurs mainly during and soon after delivery, through direct contact of the infant with infectious blood and other body fluids. Previous studies demonstrated that elective cesarean section (ECS) may reduce the risk of mother-to-child transmission of some viruses, such as human immunodeficiency virus and herpes simplex virus [2,3]. Similarly, some studies found that HBV infection rate in infants born by vaginal delivery (VD) was higher than that in infants born by ECS [4-6]. Therefore, many clinicians and HBV-infected pregnant women prefer ECS to prevent mother-to-child transmission of HBV [7], although other reports show that ECS does not reduce the likelihood of HBV infection in infants [8,9]. In this study, we compared the HBV infection rates in children of hepatitis B surface antigen (HBsAg)-positive mothers who delivered their babies by ECS or VD to clarify the issue of whether ECS may reduce the risk of mother-to-child transmission of HBV.

## Methods

### Subjects and serum specimens

In the present study, two groups of children born to HBsAg-positive mothers were recruited. The first group was composed of 296 singleton full-term children and their 296 HBV-infected mothers. These mothers were selected based on the results of a retrospective study on the prevalence of HBsAg among pregnant women from 14 cities and rural areas across Jiangsu during 2002–2004 [10]; they were all HBsAg-positive during their pregnancy and at delivery. Serum samples (~3 ml each) from these 296 children were prospectively collected from October 2009 to March 2010, when they were at 5–7 years old. The second group was comprised of 250 children aged 1–6 years who were born to 248 HBV-infected mothers (2 pairs of twins). These children were recruited based on our invitation of 385 mothers, who were all HBsAg-positive during their pregnancy and delivered their babies in Zhenjiang Fourth People's Hospital, Jiangsu, from January 2006 to December 2010, to join this study. Blood sample (~3 ml each) was collected from each child from November 2011 to March 2012.

Each attending mother was asked to complete a questionnaire, which included demographic data of the mother and her child, the use of HBIG and first dose hepatitis B vaccine in the children. These data were all further validated by checking the hospital records. In addition, the results of HBV serologic markers and HBV DNA levels in each mother at delivery were retrieved from medical records. The use of second and third doses of hepatitis B vaccine was confirmed in 90.3% of the children by checking the children's vaccination records, and that in other instances was defined by the interview with children’s mothers.

All mothers had no coinfection with hepatitis C virus and human immunodeficiency virus, and had not received amniocentesis or anti-viral treatment before and/or during pregnancy. Those with maternal complications, such as acute hepatitis and threatening abortion, were also excluded. Totally 546 children (2 pairs of twins) born to 544 HBsAg-positive mothers were enrolled. The mothers were 23–48 years of age (mean, 31.0 ± 3.5), and the gestational ages were 35–42 weeks (39.5 ± 1.0). The children (296 boys) were 1–7 years old (4.7 ± 1.7) during follow-up; 25.1% (137/546) were born to hepatitis B e antigen (HBeAg)-positive mothers.

This study was performed according to the Declaration of Helsinki and approved by the institutional review boards of Nanjing Drum Tower Hospital and Zhenjiang Fourth People's Hospital. Each mother gave the written informed consent for the use of her child’s blood samples.

### Assays for HBV serologic markers

Commercial enzyme immunoassay kits (Huakang Biotech, Shenzhen; Kehua Bio-Engineering, Shanghai, China) were used for qualitative testing of HBV serologic markers, including HBsAg, antibody against hepatitis B surface antigen (anti-HBs), and antibody against hepatitis B core antigen (anti-HBc). All HBsAg-positive specimens were also tested for HBeAg and antibody against hepatitis B e antigen (anti-HBe) by EIA (Huakang Biotech; Kehua Bio-Engineering). Quantitative analysis of anti-HBs in a serum sample was further performed using a microparticle immunoassay kit (Architect system, Abbott, North Chicago). Positive and negative controls were included in each test. All samples were tested by two technicians who were unaware of the serum identity. When anti-HBs was beyond the upper detection limit (1000 mIU/ml), the specimens were retested after further dilution with 20% bovine fetal serum in phosphate-buffer saline.

### Statistical analysis

Data analysis was performed using SPSS software version 17.0 (SPSS Standard version 17.0, SPSS Inc., Chicago, IL). Continuous variables normally distributed were expressed as mean ± standard deviation (SD) and compared by *t*-test between two groups. Quantitative data non-normally distributed were presented as median and interquartile range. Categorical variables were reported as number and percentage and compared by *χ*^2^ test or Fisher’s exact test where appropriate. A two-sided p value of <0.05 was considered as statistically significant.

## Results

### General characteristics of the study population

Of the total 546 children, 285 (52.2%) were delivered by ECS and 261 others (47.8%) were delivered by VD. They were born to 284 and 260 mothers respectively, one pair of twin in each group. The mothers of the children in the two groups had comparable maternal and gestational ages and HBeAg-positive rates (Table 1). The children in the ECS and VD groups also had similar ages, gender ratios, feeding modes, hepatitis B vaccine coverage and administrations of HBIG (Table 1). All the children were generally in good health at the follow-up.

**Table 1:** General characteristics of children and their mothers in ECS and VD groups^a^

**Characteristic**	**ECS (n = 285)**	**VD (n = 261)**	**p**
Maternal age, mean (SD)	31.3 ± 3.7	30.8 ± 3.4	0.093
Gestational age, mean (SD)	39.3 ± 0.9	39.6 ± 1.1	0.892
Mother with positive HBeAg, n (%)	67 (23.6)	68 (26.2)	0.489
Child’s age, mean (SD)	4.6 ± 1.8	4.8 ± 1.7	0.106
Male child, n (%)	152 (53.3)	144 (55.2)	0.667
Breast/formula-feeding	202/83	195/66	0.315
Timely first dose vaccine, n (%)	259 (90.9)	230 (88.1)	0.293
Hepatitis B vaccine coverage, n (%)	285 (100)	261 (100)	ND
HBIG within 24 hours after birth, n (%)	162 (56.8)	129 (49.4)	0.083

^a^ There was one pair of twin born to HBeAg-positive mother in each group.

### HBV infection in children of carrier mothers

HBV infection was defined by the positive results of HBsAg, anti-HBc, and HBeAg. The overall rate of chronic HBV infection in the children of HBsAg-positive mothers was 2.4% (13/546), although they were all vaccinated against hepatitis B and mostly administered with HBIG within 24 hours after birth. As shown in Table 2, the rate of chronic infection and self-resolved infection, presented as HBsAg-negative and anti-HBc-positive, in the VD group was 2.3% (6/261) and 3.8% (10/261) respectively, each comparable to that in the ECS group (p = 0.904 and 0.355 respectively).

**Table 2:** HBV serologic markers in 546 children of HBsAg-positive mothers who delivered their infants by ECS and VD

**Serologic marker**	**ECS (n = 285)**	**VD (n = 261)**	**p**
HBsAg+, n (%)	7 (2.5)	6 (2.3)	0.904
Anti-HBc+/HBsAg–, n (%)	7 (2.5)	10 (3.8)	0.355
Anti-HBs ≥10 mIU/ml, n (%)	206 (72.3)	185 (70.9)	0.717

It has been demonstrated that the major risk factor responsible for mother-to-child transmission of HBV is the viral load in mothers [11]. Maternal HBeAg positivity is well correlated with the HBV DNA levels [12]. Thus, infants of HBeAg-positive mothers have an increased risk of becoming infected with HBV. In agreement with the findings, all 13 infected children in the present study were born to HBeAg-positive mothers and none of the children born to HBeAg-negative mothers was chronically infected. To clarify whether ECS may reduce the likelihood of mother-to-child transmission of HBV in these high-risk children, we compared the incidence of HBV infection in 68 children of HBeAg-positive mothers who underwent ECS with that in 69 other children of HBeAg-positive mothers who delivered vaginally. The mothers who delivered their babies by ECS had comparable HBV DNA levels with those who delivered their babies by VD (2.38 × 10^6^ vs. 2.35 × 10^6^ IU/ml, p = 0.586). Similarly, as shown in Table 3, the rates of chronic infection and self-resolved infection were both comparable between the two groups (10.3% vs. 8.7%, p = 0.750 and 5.9% vs. 13.0%, p = 0.153).

**Table 3:** HBV serologic markers in 137 children of HBeAg-positive mothers who delivered their babies by ECS and VD^a^

**Serologic marker**	**ECS (n = 68)**	**VD (n = 69)**	**p**
HBsAg+, n (%)	7 (10.3)	6 (8.7)	0.750
Anti-HBc+/HBsAg–, n (%)	4 (5.9)	9 (13.0)	0.153
Anti-HBs ≥10 mIU/ml, n (%)	55 (80.9)	52 (75.4)	0.408

^a^ The mothers in ECS and VD groups had comparable HBV DNA levels (2.38 × 10^6^ vs. 2.35 × 10^6^ IU/ml, p = 0.586).

### Anti-HBs levels in children

Of the 546 children who had been vaccinated against hepatitis B, 391 (71.6%) had anti-HBs ≥10 mIU/ml, which is similar to the findings in children with same ages [13]. The anti-HBs positive (≥10 mIU/ml) rate in the 291 children who were administered HBIG at birth was not different from that in those who were not administered HBIG (74.9% vs. 67.8%, p = 0.068). Furthermore, the positive rate of anti-HBs in children born by ECS was similar to that in children born by VD, even in those born to HBeAg-positive mothers (Tables 2 and 3).

## Discussion

Our study showed that, under the currently available immunoprophylaxis against hepatitis B, children of HBsAg-positive mothers delivered by ECS and VD had a similar prevalence of HBsAg, demonstrating that ECS does not reduce the risk of mother-to-child transmission of HBV. Therefore, ECS should not be used as a measure to prevent mother-to-child transmission of HBV.

Although HBV DNA may be detected in maternal blood and other body fluids of HBV carrier mothers and ECS may shorten the time of delivery [14], we found in the present study that the HBV infection rate in the children delivered by ECS was comparable with that in the other children delivered by VD (Table 2). Since the percentage of HBeAg-positive mothers, the use of hepatitis B vaccine and HBIG in children and other general characteristics were comparable between the ECS and VD groups (Table 1), the infection rate in either group was not influenced by these factors. Additionally, the HBeAg-positive rate (24.8%) in HBV-infected pregnant women in the present study was in accordance with the rates reported in previous studies [15,16], suggesting that the study subjects were representative. Since infants of HBeAg-positive mothers are more prone to be infected [17], we further analyzed the HBV infection in the 137 children of HBeAg-positive mothers. The results demonstrated that ECS does not reduce the risk of mother-to-child transmission of HBV, even in the children born to HBeAg-positive mothers (Tables 2 and 3).

Before the availability of HBIG and hepatitis B vaccine, Chen et al. [8] described a cohort of 23 infants born by ECS and 73 infants born by VD, whose mothers were all asymptomatic chronic HBV carriers; the mother-to-child transmission was similar in infants (≥ 6 months) delivered by ECS and VD (39.1% vs. 43.8%). In addition, the rates of acquisition of HBsAg were comparable between the infants born after the first stage of labor >9 hours and ≤9 hours (41.9% vs. 45.2%). The data show that prolonged labor and uterine contractions at delivery play little role in mother-to-child transmission of HBV. Wang et al. [9] also indicated no significant effects of delivery mode on the prevention of mother-to-child transmission of HBV. Recently, using highly sensitive real-time PCR, Papaevangelou et al. [18] reported that HBV DNA in peripheral blood of newborns was more often detected than HBsAg, however, no difference in the incidence of neonatal viremia was observed between the babies born by ECS and VD (21.9% vs. 26.5%, p = 0.685). Our data in the present study are in accordance with these reported results.

Lee et al. [4] advised ECS for HBeAg-positive pregnant women because they found higher rate of HBV infection in infants delivered vaginally within 6 months of age and serum HBV DNA at birth. Similarly, a recent study in India presented higher transmission of HBV in babies delivered by vaginal route than by cesarean section [5]. However, these two studies did not follow the infants to 9–18 months old, the age point by which the perinatal infection can be defined [19]. Furthermore, although a systematic review suggested that ECS appears to be effective in preventing mother-to-child transmission of HBV [6], high risk of bias included in the analyses should not be overlooked.

It is concerning to see that the birth dose vaccine was delayed in 10.4% of the newborns and only half of the infants in the present investigation received HBIG within 24 hours after birth. The untimely use of first dose vaccine and the low rate of HBIG administration indicated that there are considerable gaps in the immunoprophylaxis against hepatitis B between the national recommendations and routine practices in China [20]. Therefore, more measures should be taken in the future to achieve full adherence to the recommended prophylaxis in preventing mother-to-child transmission of HBV.

The main limitation in our study is that the mothers’ delivery modes were not randomly assigned. However, as mentioned above, maternal and neonatal general characteristics were comparable between ECS and VD groups. Furthermore, maternal HBeAg-positive rates and HBV DNA levels in HBeAg-positive mothers were also similar between the two groups. Thus, it was less likely the non-randomized design in the present study may result in significant bias. Additionally, ethical considerations will not allow such a randomized study. The other minor flaw is that the use of second and third doses of hepatitis B vaccine in some 10% of the children was defined by the interview with their mothers. However, we consider that the data were reliable because all newborns in China have received three doses of hepatitis B vaccine without charge since 2002 [21]. Additionally, the overall HBsAg-positive rate (2.4%, 13/546) and the rate (71.6%) of anti-HBs ≥10 mIU/ml also indicate that these children had been vaccinated against hepatitis B.

## Conclusion

In conclusion, with passive and active immunoprophylaxis against hepatitis B, ECS does not reduce the risk of mother-to-child transmission of HBV in infants of HBV carrier mothers. Therefore, ECS should not be recommended in HBsAg-positive pregnant women to prevent mother-to-child transmission of HBV.

## Abbreviations

HBV: Hepatitis B virus; HBIG: Hepatitis B immune globulin; ECS: Elective cesarean section; VD: Vaginal delivery; HBsAg: Hepatitis B surface antigen; HBeAg: Hepatitis B e antigen; anti-HBs: Antibody against hepatitis B surface antigen; anti-HBc: Antibody against hepatitis B core antigen.

## Competing interests

The authors declare that they have no competing interests.

## Authors’ contributions

YHZ was responsible for the conception and design of the study, and participated in interpretation of the data and revision of the manuscript. YH participated in the study design, data collection and interpretation, and wrote the first draft. JC performed the experiments, interpreted relevant results, and drafted the manuscript. JW carried out the data acquisition, statistical analysis and interpretation of the data, and assisted in drafting the manuscript. CX made substantive contributions to data collection, conducted the experiments, and commented on the manuscript. SZ participated in data collection and analysis, assisted in drafting the manuscript. BX was responsible for the statistical analysis and interpretation of the results, and participated in revising the manuscript. All authors read and approved the final manuscript.

## Pre-publication history

The pre-publication history for this paper can be accessed here:

http://www.biomedcentral.com/1471-2393/13/119/prepub
